# Emotions in motion: How fear and anger differently influence forward single-step initiation

**DOI:** 10.1007/s00426-026-02317-2

**Published:** 2026-06-04

**Authors:** Laure Coudrat, Stéphanie Caharel, Jean-Luc Kop, Loïc P. Heurley

**Affiliations:** 1 Univ Toulon, J-AP2S, CS60584, Campus de la Garde, Toulon, 83041 Cedex 9 France; 2 Université de Lorraine, UR 7489, 2LPN-CEMA Group (Cognition-EMotion-Action), BP3142, 91 avenue de la libération, Nancy cedex, 54021 France; 3 Université de Lorraine, INTERPSY, Nancy, F-54000 France; 4 LICAE (Laboratoire sur les Interactions Cognition, Action, Emotion), Université Paris Nanterre, 200 avenue de la République, Nanterre, 92000 France; 5 Laboratoire EPSYLON EA 4556, route de Mende, Université de Montpellier Paul Valéry, Montpellier, 34090 France

## Abstract

The perception of emotional faces plays a critical role in shaping human motor responses. However, the relationship between perceived facial expressions, particularly fear, and approach-related movement remains unclear as prior research often employed ambiguous approach behaviour and tasks requiring emotion identification before action planning, potentially introducing biases from differences in recognition accuracy across emotions. To address these limitations, we examined the effect of emotional (fearful, angry, happy) faces on unambiguous approach-related movement, i.e., forward single-step initiation by using a go/no-go paradigm, in two conditions: a neutral-versus-emotional condition (motor responses depended on whether the face was emotional or neutral), or a face-gender condition. Results showed that the perception of emotional faces impacted forward single-step initiation in the neutral-versus-emotional condition only. More precisely, the motor response was stronger (higher anticipatory postural adjustments amplitude) in front of fearful and happy faces than angry faces, and faster (shorter time to reach the forward velocity peak of the centre of body mass) to fearful than to angry faces. This modulation of approach-related motor response, with facilitation of motor output for fearful relative to angry faces, may reflect a more effective preparation of forward stepping when the emotional information is compatible with the movement. Furthermore, the fact that emotional effects were observed only in the neutral-versus-emotional condition can be interpreted in terms of whether the emotional information was task-relevant or incidental.

## Introduction

Emotion and action are two fundamental components of human behaviour that are intimately linked. The biphasic theory of emotion (Bradley et al., 2001; Lang et al., 1990) is classically used to explain the impact of emotion on motor output associated with goal-directed movement. According to this theory, emotion is fundamentally organized around two basic motivational systems: the appetitive system that induces approach behaviours and the defensive system priming avoidance behaviours. Whereas the appetitive motivational system is thought to be activated in pleasant contexts (i.e., contexts that promote survival), the defensive motivational system is thought to be activated in unpleasant ones (i.e., contexts involving threat). In line with this motivational direction hypothesis, it has been found that approach/avoidance behaviours associated to pleasant/unpleasant stimuli, respectively, are facilitated as compared to unpleasant/pleasant stimuli, respectively (e.g., Chen & Bargh, 1999; Eder & Rothermund, 2008; Seibt et al., 2008). This was originally shown in Chen and Bargh (1999) study in which participants performed a motor task requiring pulling (i.e., upper limb flexion) or pushing (i.e., upper limb extension) a lever according to the valence of a word that appeared on a screen placed in front of them. Importantly, in this setup, the flexion or extension of the arm was conceptualized as approach or avoidance behaviour, respectively. The results showed that movement performance was enhanced when the direction of the goal-directed movement (i.e., pulling/approach or pushing/avoidance) was congruent with the behavioural direction induced by the emotional word (i.e., pleasant/approach, unpleasant/avoidance). More precisely, faster response times were observed for pulling rather than pushing in response to pleasant words and for pushing rather than pulling in response to unpleasant words. These results supported the idea that pleasant and unpleasant stimuli facilitate approach and avoidance goal-directed upper-limb movements through the activation of the appetitive and defensive motivational systems, respectively.

Moreover, using a protocol similar to that of Chen and Bargh (1999) study but with emotional faces, Marsh et al. (2005a) showed that participants performed better when pushing rather than pulling in response to anger and when pulling rather than pushing a lever in response to fear. These results suggested that while anger would prime avoidance behaviour from perceiver, indicating that this negative expression is indeed perceived as threatening, fear, on the contrary, would prime approach behaviour, insinuating that this expression would be perceived as appealing and not as aversive (Marsh et al. 2005a). These findings challenge the view that angry expression is predominantly appetitive (e.g. Carver et al., 2009; Wilkowski & Meier, 2010). For example, in the study by Wilkowski and Meier (2010), based on a variant of Chen and Bargh (1999) task (see also, for the use of a similar paradigm, Markman and Brendl (2005), participants categorized emotional faces by moving an on-screen representation of themselves either toward or away from the emotional stimulus using a push-pull lever. On-screen movements in which participants approached angry faces with their self-representation were initiated more rapidly than movements directed away from them, regardless of whether the action involved pushing or pulling the lever (Wilkowski & Meier, 2010). In contrast, movements away from fearful faces tended to be initiated faster than movements toward them, although this effect was not statistically significant (Wilkowski & Meier, 2010).

The discrepancy between these opposite patterns of results may be attributable, at least in part, to the ambiguity of the lever-based paradigm. It has been pointed out that pulling or pushing a lever could be ambiguous as to whether it represents an approach or avoidance movement (e.g. Eder & Rothermund, 2008; Markman & Brendl, 2005; Seibt et al., 2008). In their study, Seibt et al. (2008) used two opposite conceptualizations of lever movements. According to the first one, in which the frame of reference was the self, pulling or pushing a lever was defined as approach or avoidance behaviour: executing a toward (pulling) or an away-from (pushing) participant movement. In contrast, according to the second one, in which the frame of reference was the object (i.e., a word that appeared on a screen in front of the participant), pulling or pushing a lever was conceptualized as avoidance or approach behaviour: executing a toward (pushing) or an away-from (pulling) object movement. The results showed that when the frame of reference was the self, participants performed better when pulling for positive words and when pushing for negative words. In contrast, when the frame of reference was the object, participants performed better when pushing for positive words and when pulling for negative words. It therefore appears that pulling or pushing a lever can be influenced in opposite ways by a pleasant or unpleasant stimulus, depending on the conceptualization adopted. Thus, given that Marsh et al. (2005a) used a potentially ambiguous motor task (i.e. pulling or pushing a lever), it remains unclear whether the perception of fear or anger promotes approach or avoidance movement, respectively.

Interestingly, the initiation of forward single or multi-step walking is defined as an unambiguous approach movement, as it clearly decreases the distance between the self and the affective stimulus location (Strack & Deutsch, 2004). This transition phase between the quiet standing posture in double limb support and the single/multi-step pattern involves complex posture-movement coordination known as anticipatory postural adjustments (APAs). Investigating APAs provides valuable insight into the mechanisms of postural control in both healthy and clinical populations. In addition, the fact that this whole-body movement is daily performed to interact with the environment makes it more ecological than pulling or pushing a lever. According to the biphasic theory of emotion (Bradley et al., 2001; Lang et al., 1990), some studies showed that this whole-body goal-directed movement was slowed down, as attested by longer reaction time and time to reach the forward velocity peak of the centre of mass, when participants had to initiate the movement as soon as possible after the onset of an unpleasant picture, as compared to a pleasant one (these pictures representing different human experiences) (e.g. Gélat et al., 2011; Stins & Beek, 2011; Yiou et al., 2014). Notably, Gélat et al. (2011) also reported a weaker motor response, as evidenced by smaller amplitudes of APAs for unpleasant versus pleasant pictures. Importantly, the impeding of this whole-body approach movement in unpleasant context could stem from an emotional conflict between the motivational system engaged by the emotional stimuli (i.e., defensive) and the direction of the goal-directed movement (i.e., approach; Gélat et al., 2011). Moreover, using facial expressions, it was reported that forward single step was initiated slower (i.e., longer reaction time) towards an angry face than towards a happy face (Mirabella et al., 2023; Stins et al., 2011, 2014). This modulation of approach-related motor responding in the presence of angry facial expressions may suggest that such stimuli engage an avoidance-oriented motivational process. Nevertheless, as these previous studies focused only on happy and angry expressions, it is unclear whether fear activates the appetitive motivational system. To our knowledge, only one study has examined forward single-step initiation in response to fearful and angry faces (Lebert et al., 2024). Their results showed that reaction time was longer when initiating the forward step in response to fear compared to anger. The slowing down of the approach-related motor responding in front of fear, compared to anger, may suggest that these stimuli differentially engage avoidance- and approach-oriented motivational processes, a pattern that is consistent with the view according to which anger is associated with approach and fear with avoidance (e.g. Carver et al., 2009). However, in this study (Lebert et al., 2024), it is important to note that the task required participants to execute or withhold the movement (go/no-go task) based on their identification of the emotion conveyed by the face, specifically distinguishing between fear and anger. Indeed, in one condition, participants had to move or not if the face expressed anger or fear, respectively. In the other condition, the instruction was reversed. Given that fear is usually recognized less accurately than other emotions, such as anger (e.g. Palermo & Coltheart, 2004), the longer reaction time to initiate a forward single step in front of fearful face may result from an extended processing time to identify fearful, compared to angry faces.

Taking these limitations into consideration, the main objective of the present study was to better understand the complex interplay between emotion and action and more specifically, to clarify the influence of fearful and angry expressions on the spatiotemporal parameters of an unambiguous approach movement, i.e., forward single step initiation. For these purposes, a go/no-go paradigm was used during which participants performed a single step towards (i.e., approach) happy, angry, or fearful faces, without needing to distinguish between these three emotional faces to produce the movement. According to the results of Marsh et al. (2005a), we expected a facilitating effect of happy and fearful faces compared to angry faces on the motor output of the whole-body approach movement, with facilitation primarily characterized by reduced reaction time and time to reach the peak of centre of mass velocity, together with increased APAs amplitude. Moreover, in the meta-analysis of Phaf et al. (2014), the instruction (i.e., whether or not to attend to stimulus valence) was identified as the most important moderator of the relationship between emotional stimuli and approach/avoidance movements. More precisely, whereas the emotional effect on approach/avoidance movements occurs when the instructions require explicit processing of the stimulus valence (i.e., explicit condition), this effect disappears when the instructions do not require such processing (i.e., implicit condition), where the terms “explicit” and “implicit” are those used by the authors of the cited article. In addition, the findings that emotional stimuli influence motor behaviour only when task-relevant, and not when task-irrelevant, have been confirmed in a subsequent review (Mirabella & Montalti, 2025). Taken together, these results suggest that the process of emotional stimuli is not automatic, in the sense that they do not impact the motor output when they are task-irrelevant or when the instruction do not require their processing. However, these studies have primarily examined emotion-action relationships in tasks involving only upper-limb movements, such as those involved in pushing or pulling a lever. To our knowledge, only one study has extended these findings to a complex whole-body movement, such as forward single-step initiation (Mirabella et al., 2023), although it did not consider fearful faces. Thus, the second aim of the present study was to test the interaction between emotion and condition on the motor output of forward single-step initiation. To this end, participants performed two conditions. In the neutral-versus-emotional condition, they had to move (i.e., go trials) or not (i.e., no-go trials), depending on whether the face was neutral (i.e., no-go trials) or not (i.e., go trials). In this condition, the task was designed to ensure that the emotional content conveyed by the face was processed (i.e., the presence versus absence of emotion). In the face-gender condition, they had to move or not according to the gender of the face. In this condition, although all faces conveyed emotional expressions, the emotional information was incidental. According to Phaf et al. (2014) and Mirabella and Montalti (2025), we predicted a facilitating effect of happy and fear expressions on forward single step in the neutral-versus-emotional condition only.

## Method

### Participants

To define our sample size, we relied on the study by Stins et al. (2014) using a paradigm similar to ours. In their study, an effect of emotional faces (happy, neutral and angry) was observed on the reaction time to initiate a single step in a condition in which only a small effect size was expected (i.e., face-gender condition). We therefore sought to determine whether a similar sample size (*n* = 24) provided adequate power to test our hypotheses. However, because Stins et al. (2014) did not examine the amplitude of APAs along the anteroposterior axis, which is the primary variable of the present study, we based our power analysis on the mean and standard deviation values reported by Gélat et al. (2018). Power analysis was performed with R package “simr” (Green & MacLeod, 2016). Fixed effects parameters were estimated based on the APAs-related results by Gélat et al. (2018), assuming a small effect size corresponding to a Cohen’s d coefficient of 0.25. By setting the number of simulations to 1000, the estimated power was 99.80%, with a 95% confidence interval of 99.28 to 99.98. The power of the study was therefore sufficient with the parameters as they were defined. The detailed strategy and main lines of code for power analysis are available from the Open Science Framework platform (https://osf.io/a5439/overview).

Thus, twenty-four healthy young adults (age 18–31 years, 12 women) volunteered for this study which was carried out in 2018. All had normal or corrected-normal vision, no current lower extremity injury and were unaware of the purpose of the study. Each participant signed an informed consent before the experimental procedures. The study complied with the Helsinki Declaration for human experimentation and was approved by the local ethics committee.

### Experimental design

Participants stood barefoot at the back of a force platform (AMTI, 120 × 60 cm) placed 2.80 m in front of a white board of the laboratory on which emotional faces (straight angle) were presented. The positioning of the feet allowed the single step to be performed on the platform and was marked to be used in all trials.

**Fig. 1:**
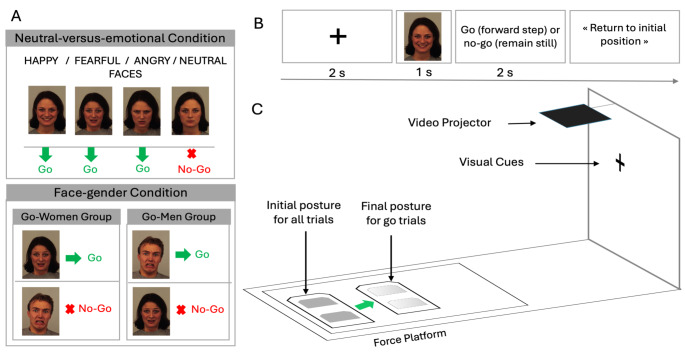
(**A**) Examples of the emotional faces used in the neutral-versus-emotional and face-gender conditions. (**B**) Temporal sequence of visual cues. (**C**) Postural setup. The KDEF stimuli included in this figure are as follows: AF01HAS, AF01AFS, AF01ANS, AF01NEA, AM08AFS

Using a go/no-go reaction time paradigm, the task consisted in achieving a forward single step with the dominant leg (Fig. 1C). The dominant leg was determined using classical motor tasks including ball-kicking and spontaneous step initiation (Yiou et al., 2014).

Two experimental conditions were tested in a random order. In the neutral-versus-emotional condition, participants were asked not to move (i.e., no-go response) if the emotional expression conveyed by the presented face was judged as neutral. In contrast, they were instructed to initiate a forward single step (i.e., go response) if the facial emotional expression was judged as happy, angry, or fearful. In the face-gender condition, the participants judged whether the face was a male or a female regardless of the emotional facial expression. Half of the participants were asked to initiate a single step for female faces and not to move for male faces (go-women group). The other half received the reverse instruction (go-men group) (Fig. 1A). All participants underwent a training phase before being tested.

### Emotional stimuli

All facial stimuli used in the present study were selected from the Karolinska Directed Emotional Faces (KDEF; Lundqvist et al., 1998) database, a widely validated set of facial expressions (Goeleven et al., 2008). For each face identity (13 men and 12 women), four emotional expressions were selected, namely neutral, fearful, angry and happy. All faces were presented in frontal view, with a direct gaze. To ensure that the selected facial expressions were clearly and reliably recognized, a preliminary study was conducted with a separate group of 10 healthy participants (age 18–42 years, 3 women), who had to categorize expressions through an eight-alternative forced-choice task (neutral, fearful, angry, happy, disgust, surprise, sadness, and unknown). In the end, the 10 faces^1^ (5 men and 5 women) that were best recognized across all four expressions (i.e., with a recognition rate greater than 80%) were selected for the main experiment.

During the main experiment, each trial began with the presentation of a central fixation cross on the screen for 2 s, followed by a face display for 1 s. A white screen then appeared until the end of the movement (Fig. 1B).

In each condition, the faces were presented in a random order. In the neutral-versus-emotional condition, the 10 faces (5 men and 5 women), each conveying the four emotional expressions - neutral (no-go trials), fearful, angry, and happy (go trials) were displayed. In the face-gender condition, the same previous faces displaying only expressions of fear, anger, and happiness were presented. For the go-men group, the 5 faces of man displaying the 3 emotional expressions were presented two times (go trials). In addition, 3 faces of women displaying the 3 expressions and one face of woman displaying anger were presented one time (no-go trials). A similar design was applied for the go-women group. In total, for each condition, participants performed 30 go and 10 no-go trials (Table 1). Thus, the neutral-versus-emotional and face-gender conditions differed not only in the task-relevance of emotional expressions but also in response mapping and decision criteria, as participants categorized faces based on emotion in the neutral-versus-emotional condition and based on gender in the face-gender condition.

**Table 1:** Composition of go and no-go trials across experimental conditions and groups

Condition	Group	Stimuli	Trial type	Emotional expression	Number of trials
Neutral-versus-emotional	All participants	10 faces (5 men, 5 women)	Go	Fearful, Angry, Happy	30
			No-go	Neutral	10
Face-gender	Go-men group	5 male faces	Go (2 repetitions)	Fearful, Angry, Happy	30
		4 female faces	No-go	Fearful, Angry, Happy (3 faces); Angry only (1 face)	10
	Go-women group	5 female faces	Go (2 repetitions)	Fearful, Angry, Happy	30
		4 male faces	No-go	Fearful, Angry, Happy (3 faces); Angry only (1 face)	10

The male and female faces of the face-gender condition are the same as those used in the neutral-versus-emotional condition

The Psychophysics Toolbox Version 3 (Brainard, 1997) on Matlab R2019b was used to control the visual stimuli presentation of each trial and its synchronization with the signals of the force platform.

### Data reduction

All signals from the force platform were recorded at 1000 Hz. These signals were filtered with a 10 Hz low-pass, fourth order, zero-lag Butterworth filter. Coordinates of the centre of pressure (CP) on the antero-posterior (AP) direction (yP) were calculated from the ground reaction forces and moments measured by the force platform. The displacement of CP on AP direction was derived to obtain the CP velocity (y’P).

The body’s centre of mass (CM) acceleration on AP axis (y’’G) was obtained from Newton’s law and the CM velocity (y’G) axis was calculated by simple integration, by posing that y’’G and y’G equaled zero at movement onset (i.e., t0y). That enabled the calculation of the peak value of forward CM velocity reached during the single step.

### Outcome variables

**Fig. 2:**
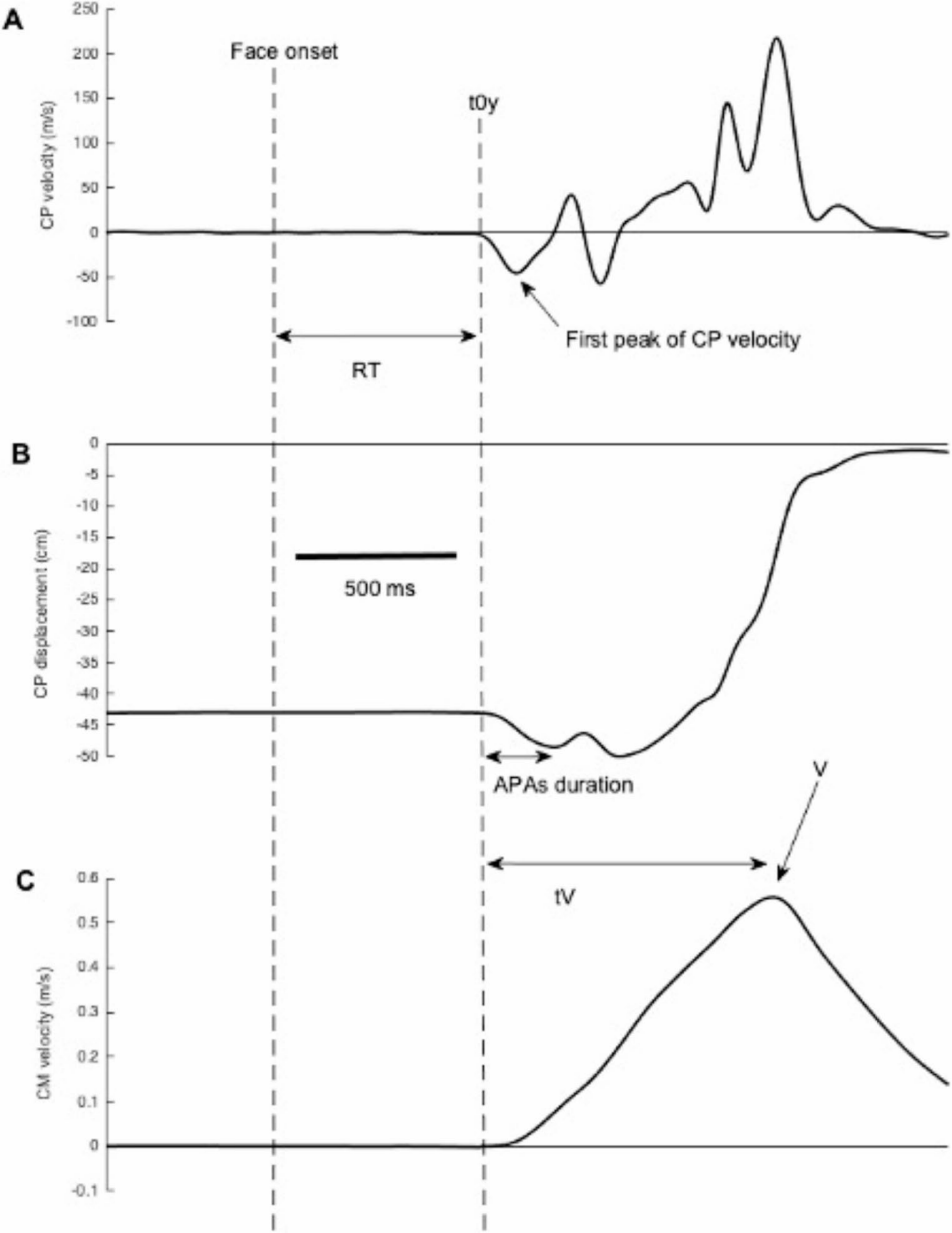
(**A**) anteroposterior velocity of centre of pressure (CP); (**B**) anteroposterior displacement of CP; (**C**) anteroposterior velocity of centre of mass (CM); one trial, one subject, during a forward single step in front of a happy face in the face-gender condition. From the top to bottom, the horizontal arrows correspond to the reaction time (RT), the duration of anticipatory postural adjustments on the anteroposterior axis (APAs) and the time to reach the peak of forward CM velocity (tV)

Six outcome variables were studied. The initial posture corresponded to the mean CP value on AP direction computed during the 500 ms time window before face onset. A higher value indicated a centre of pressure further ahead. The onset of postural modifications along the AP axis (t0y) was calculated as the time at which 10% of the first peak of y′P was reached, as in previous studies (Gélat et al., 2011, 2018). The reaction time corresponded to the time delay between the onset of the emotional face and t0y. The APAs duration along the AP axis was defined as the delay from t0y to the time the first peak of backward shift of CP was reached. The APAs amplitude corresponded to the initial backward shift of CP. The motor performance was quantified through the analysis of the peak of forward CM velocity (V) and the time when it occurred (tV; Fig. 2).

### Statistical analysis

On the 1440 go trials that were collected, 35 trials (2.5%) were rejected because either (a) a no-go response was produced, (b) a step was made with the wrong limb, (c) the reaction time fell outside the range 200–2000 ms, or (d) a considerable CP movement occurred during the 500 ms window prior to face onset.

All the statistical analyses were performed with R statistical software (R Core Team, 2020). Linear mixed model analyses were performed with R package “lme4” (Bates et al., 2015). The mixed model approach was chosen over generalized linear model (repeated-measure analysis of variance [ANOVA]) because it is adapted to repeated measurements by taking into account crossed random effects such as participants and items (Baayen et al., 2008; Judd et al., 2012). The distributions of all outcome variables were first examined by inspecting skewness and kurtosis values and no substantial deviations from normality were observed. Subsequently, each study variable was analysed using a mixed-model with emotion (fear, angry, happiness), condition (neutral-versus-emotional, face-gender) and the interaction between emotion and condition as fixed factors and with random intercepts for both participants and faces. The significance of the effects was obtained using R package “lmerTest” (Kuznetsova et al., 2017), in which the number of degrees of freedom is estimated using the Satterthwaite method. Least-squares mean calculations and post hoc comparisons were performed with R package “emmeans” (Lenth, 2020), with Tukey adjustment for *p*-value adapted for multiple comparisons. For all analyses, the probability was set at *p* < 0.05. Effect sizes were calculated as R^2^ estimated according to the Kenward-Roger method (Edwards et al., 2008).

## Results

### Reaction time

The results of the mixed model only revealed a significant main effect of condition (*F*_(1,1359.6)_ = 51.48, *p* < 0.001, R^2^ = 0.02). The participants were faster in the face-gender than in the neutral-versus-emotional condition (Table 2).

**Table 2:** Estimated marginal mean scores on the spatiotemporal parameters of step initiation in the face-gender and neutral-versus-emotional conditions

	Face-gender	Neutral-versus-emotional	*p* ^1^
**Reaction time (ms)**	604 (13)	637 (13)	***
**Initial posture (cm)**	−44 (0.35)	−43.9 (0.35)	*
**APAs duration (ms)**	265 (9)	250 (9)	***
**APAs amplitude (cm)**	− 4.57 (0.22)	− 3.97 (0.22)	***
**tV (ms)**	948 (28)	994 (28)	***
**V (m/s)**	0.563 (0.024)	0.563 (0.024)	NS

Values in parentheses are standard errors. *p* p value, ***: *p* < 0.001, *: *p* < 0.05, NS: not significant

^1^ *S*ignificance level for the difference between conditions

### Initial posture

A significant main effect of condition was also found on initial posture (*F*_(1,1376)_ = 8.62, *p* < 0.01, R^2^ = 0.001). The results indicated that the CP position was further ahead in the neutral-versus-emotional than in the face-gender condition (Table 2). The other effects were not significant (*p* > 0.05).

### APAs duration

**Fig. 3:**
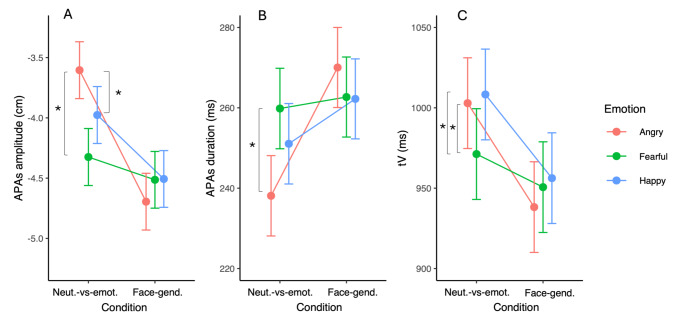
Estimated marginal mean (and standard error) scores on the Anticipatory Postural Adjustments (APAs) amplitude (**A**), APAs duration (**B**) and time for reaching the peak of forward centre of mass velocity (tV) (**C**) across emotional faces and conditions. Asterisk indicates a significant difference between emotions within the same condition (post-hoc tests). Neut.-vs-emot.: Neutral-versus-emotional; Face-gend.: Face-gender

The mixed model for APAs duration revealed a main effect of condition (*F*_(1,1361.3)_ = 13.69, *p* < 0.001, R^2^ = 0.092) indicating that it was longer in the face-gender than in the neutral-versus-emotional condition (Table 2). The main effect of emotion was not significant. A significant condition by emotion interaction (*F*_(2,1367.5)_ = 3.96, *p* = 0.013, R^2^ = 0.001) was also found. Exploration of this interaction revealed that the only significant difference was between anger and fear. More precisely, APAs duration was reduced for angry (m = 238 ms, se = 10) compared to fearful (m = 260 ms, se = 10) faces (t = −2.67, *p* = 0.025) in the neutral-versus-emotional condition only (Fig. 3B).

### APAs amplitude

The results showed that APAs amplitude was larger for the face-gender than for the neutral-versus-emotional condition (*F*_(1,1362.2)_ = 53.50, *p* < 0.001, R^2^ = 0.021) (Table 2). No main effect of emotion was observed, whereas the interaction was significant (*F*_(2,1362.4)_ = 10.16, *p* < 0.001, R^2^ = 0.05). The APAs amplitude was larger with fearful (t = 4.67, *p* < 0.001) and happy (t = 2.41, *p* = 0.047) relative to angry faces in the neutral-versus-emotional condition only (Fig. 3A). The other differences were not significant (*p* > 0.05).

### Motor performance

Amplitude in the peak of CM velocity (V) did not show any significant main effect or interaction (*p* > 0.05). Nevertheless, all fixed effects were significant for the time for reaching this peak (tV). The main effect of condition (*F*_(1,1376)_ = 91.32, *p* < 0.001, R^2^ = 0.04) indicated that it was shorter in the face-gender than in the neutral-versus-emotional condition (Table 2). The main effect of emotion (*F*_(2,1376)_ = 6.64, *p* = 0.0013, R^2^ = 0.003) revealed a shorter tV for fearful (m = 961 ms, se = 28) than for happy (m = 982 ms, se = 28) faces (t = − 3.63, *p* = 0.003). Exploration of the interaction (*F*_(2,1376)_ = 7.49, *p* < 0.001, R^2^ = 0.003) revealed that tV was shorter with fearful compared to angry (t = 3.78, *p* < 0.001) and happy (t = − 4.42, *p* < 0.001) faces in the neutral-versus-emotional condition (Fig. 3C). The other differences were not significant (*p* > 0.05).

## Discussion

The main aim of the present work was to gain a deeper understanding of the intimate link between emotion and action. More specifically, we sought to clarify the relationship between approach-related movement and the perception of emotional facial expressions, particularly fear.

To lift ambiguities noted in the existing literature, we adopted two methodological refinements. First, we used a whole-body approach movement, i.e., forward single-step initiation, which provide a clearer operationalization of approach than the lever task commonly employed. Second, we designed the task so that participants were not required to distinguish between emotional expressions (i.e, angry, fearful and happy faces) to plan and program the forward movement, thereby reducing potential biases due to differences in recognition accuracy, particularly the difficulty in identifying fear compared to anger (Palermo & Coltheart, 2004). Data gathered bring important contributions further addressed below.

### Motor responses to emotional faces in the neutral-versus-emotional condition

In the neutral-versus-emotional condition, the perception of emotional faces impacted the motor output associated to a whole-body approach movement. The most striking result of the present study highlighted a motor strategy adaptation in response to fearful faces compared to angry faces, such that the motor strategy for fear appeared to be facilitated than for anger. More specifically, the longer duration of APAs in response to fearful faces likely allowed for a sufficiently greater APAs amplitude, i.e. a larger backward shift of the CP during this movement phase. This more efficient anticipatory postural strategy for fear, compared to anger, likely contributed to improved overall movement performance, i.e. by speeding up the single-step initiation, as evidenced by a reduction in the time for reaching the peak of CM velocity, without, however, impacting the amplitude of CM velocity peak. Thus, the present findings obtained using a forward stepping task are consistent with previous results reported in a lever-based task (Marsh et al. 2005a), and point to a modulation of motor response as a function of emotional expression. One possible account is that approach-related motor responses were facilitated for fearful faces, compared to angry faces, potentially reflecting more effective preparation of forward stepping when the emotional information is compatible with the movement. In contrast, angry faces may introduce a conflict between the requirement to initiate a forward (approach-related) movement and the processing of threat-related cues conveyed by this emotional expression. This pattern may be interpreted in light with previous findings (e.g. Hammer & Marsh, 2015; Horstmann 2003; Marsh et al. 2005b; Mennella et al. 2020; Seidel et al. 2010) suggesting that angry faces are perceived as threatening and have been associated with avoidance-related tendencies, whereas fearful faces, although signalling threat, have been linked to approach-related tendencies. For example, when participants were asked to judge the number of forward or backward steps they would take if they were standing in front of a face expressing anger, sadness, disgust or happiness, participants were most willing to move backward in front of faces expressing anger (whereas they were willing to move forward only for faces expressing happiness) (Seidel et al., 2010). Moreover, while smiling faces were perceived as having an affiliative function, angry faces indicated the intention to aggress and called for backing off (Horstmann, 2003). In addition, by using a social free-choice paradigm, the proportion of avoidance responses was shown to be greater for anger than for fear (Mennella et al., 2020). Interestingly, fear faces, have sometimes been associated with approach-related responses, possibly due to their association with child faces, which also elicited approach (Hammer & Marsh, 2015). Consistent with this view, fear faces were associated with traits representing dependence, weakness, submissiveness, and warmth, whereas angry faces were judged to be associated with traits relating to maturity, strength, and dominance (Marsh et al. 2005b). Taken together, these findings provide a theoretical framework for interpreting the present results. However, given that the current task only assessed approach-movement without directly measuring motivational states, the observed facilitation of motor responses to fearful faces and related degradation for angry faces should be interpreted with caution, as reflecting a modulation of approach-related motor processes rather than direct evidence of approach or avoidance behaviours per se.

Although our findings corroborate previous evidence associating fear and anger with approach and avoidance behaviour, respectively (Marsh et al. 2005a), they deviate from those suggesting that, unlike fear, anger would prime approach behaviours through the activation of the appetitive motivational system, as it is the case for positive emotions such as happiness (for a review, see Carver & Harmon-Jones, 2009). According to this view, when participants were engaged in autobiographical emotion recall, the initiation of forward multi-step (i.e., approach) was facilitated (i.e., greater CP displacement during APAs or step length) for happiness and anger, compared with sadness and fear (Fawver et al., 2014). As already suggested by Coombes et al. (2007), it seems important to make a distinction between viewing a given basic emotion on a face and directly experiencing this specific emotion. More precisely, it could be that perceiving fear and anger on a person’s face promotes approach and avoidance responses (toward that person, as in our study), respectively, whereas directly experiencing fear and anger (e.g. through autobiographical emotion recall) preferentially drives avoidance and approach, respectively.

Furthermore, in the neutral-versus-emotional condition of our study, participants had to identify whether the face presented to them was neutral (no-go response) or emotional (go response). Importantly, the task was designed to ensure that the emotional content conveyed by the face was processed without requiring participants to distinguish between the three emotions (anger, fear, happiness) to produce the movement, in the sense that the same motor response had to be performed for all three emotional expressions. We believe this point to be fundamental. Indeed, given that fear is generally less easily identifiable than other emotions, such as anger (e.g., Palermo & Coltheart, 2004), biases could have been introduced if the motor response required distinguishing between fear and other emotions (happiness, anger).

However, the results of our study showed contrasted findings when the movement was performed in front of happy faces. Indeed, the more effective anticipatory postural strategy observed for happy faces, compared to angry faces, did not lead to an improvement of overall movement performance for happiness. The modulation of APAs in response to happy faces concerned amplitude only, and not duration. It is therefore possible that, since APAs duration did not differ between happy and angry faces, the greater APAs amplitude for happy faces was not sufficient to significantly impact overall movement performance. A possible explanation for the contrasting results for happy faces is that positive emotions, including joy, may elicit *nonspecific activation* than to negative emotions (Fredrickson, 1998). In the model of a subset of positive emotions presented by Fredrickson (1998), positive emotions are thought to broaden individuals’ scope of action, thereby promoting more diverse and less stereotyped behavioural responses, compared to negative emotions. This model, supported by empirical findings from tasks involving creativity, variety-seeking in adults, and play in children, suggests that joy may be associated with a broader range of possible responses. Such variability contrasts with the more rapid and stereotyped responses typically associated with negative emotions, particularly fear. In this regard, neurophysiological studies have shown that the amygdala plays a key role in the processing of emotional stimuli, particularly those conveying threat, such as fearful expressions (e.g., LeDoux, 1996). The processing of fearful faces is thought to rely, at least in part, on a subcortical pathway to the amygdala, enabling rapid detection of threat-related information (Méndez-Bértolo et al., 2016), even in the absence of conscious awareness (Wang et al., 2023), which may facilitate rapid adaptive responses. In this context, the urgency associated with fear may contribute to a more consistent pattern of motor responses, whereas happy faces, which convey affiliative signals, may not require such rapid responding and may therefore give rise to greater variability in motor behaviour.

Moreover, our results indicated that movement was faster/larger or slower/weaker depending on the emotional expression, suggesting that motor output is modulated by affective cues in neutral-versus-emotional condition. However, these spatiotemporal variations cannot be straightforwardly interpreted in terms of approach or avoidance tendencies. For instance, the inclusion of a neutral baseline condition might have provided a clearer reference point to determine whether changes reflected approach- or avoidance-related processes. Yet, establishing such a baseline is challenging, as it is difficult to identify a truly emotion-free condition while maintaining a similar methodology (e.g. go/no-go paradigm, neutral-versus-emotional condition that does not require distinguishing between fear and anger to plan the movement).

### The main effect of condition (face-gender versus neutral-versus-emotional)

In the present study, a condition effect (face-gender versus neutral-versus-emotional) was observed on the initial posture, i.e. on the position of the centre of pressure during the waiting phase preceding the onset of the emotional face. More precisely, the centre of pressure’s position was further ahead in the neutral-versus-emotional condition than in the face-gender one, reflecting a greater forward leaning of the whole-body when participants were asked to process the emotional content of the forthcoming emotional face. A similar forward leaning of the whole-body was already shown during the waiting phase of fast gait initiation (Lepers & Brenière, 1995), suggesting that these changes in initial posture create favourable conditions for rapid gait. In our study, however, despite favourable initial posture in the neutral-versus-emotional condition, the single step initiation was slowed down when the emotional face appeared, as attested for example by the increase of reaction time in the neutral-versus-emotional condition regardless of the valence of the emotional face. Thus, in the neutral-versus-emotional condition, it could be argued that participants did not take advantage of the favourable change in the initial posture once the emotional face has appeared. This could be explained by a general attentional capture of emotional face, which has been observed regardless of their valence (Hodsoll et al., 2011), probably interfering with the high attentional demands for walking (e.g. Lajoie et al., 1993).

### Lack of emotional influence on reaction time in the neutral-versus-emotional condition

In the present study, no effect of emotional expression was observed on reaction time in the neutral-versus-emotional condition. At first glance, this result may appear surprising, given that previous studies have reported an effect of emotion on this key variable of interest (e.g., Mirabella et al., 2023; Lebert et al., 2024). However, it is important to note that these studies differ in how reaction time is operationally defined. In the present study, the end of reaction time was defined as the onset of APAs, which was detected early through centre-of-pressure velocity. In contrast, in Mirabella et al. study (2023)—where APAs could not be assessed— the end of reaction time was defined as the onset of heel movement, which occurs after the onset of APAs. For example, according to some studies, the end of APAs corresponds to heel-off (e.g., Yiou & Do, 2010).

Furthermore, although reaction time, as well as the duration and amplitude of APAs, represent three distinct variables of interest, they are functionally interrelated. The preparation of the motor command associated with APAs during single-step initiation has been investigated using a loud acoustic stimulus (MacKinnon et al., 2007). The results of this study indicated that the spatiotemporal characteristics of APAs are progressively specified prior to movement onset. In the present study, to prevent participants from preparing their motor response in advance (i.e. before the onset of the face), the go/no-go paradigm was designed with a proportion of no-go trials (i.e., 25%) intended to limit anticipatory motor preparation (Carlsen et al., 2008). This methodology allowed us to examine the impact of emotional faces on the construction of the motor command. Thus, the emotional effect obtained on motor parameters in the neutral-versus-emotional condition suggests that the motor program was differentially programmed as a function of emotional face during the reaction time phase, without affecting its duration. Therefore, the absence of an effect on reaction time does not necessarily imply the absence of an influence on motor behaviour.

### Lack of emotional influence on motor response in the face-gender condition

In the present study, the perception of emotional faces impacted the whole-body approach movement in the neutral-versus-emotional condition only. When the instruction required participants to attend and judge the gender of the emotional face (i.e., face-gender condition), the emotional effect observed on the movement parameters no longer appeared. This result is consistent with previous data obtained in tasks involving upper-limb movements (Mirabella & Montalti, 2025; Phaf et al., 2014), where emotional stimuli influenced motor output depending on whether their valence was task-relevant. It also aligns with data from a study using a whole-body forward movement task (Mirabella et al., 2023) that was closely related to ours, which examined happy and angry faces but did not include fearful expressions. By investigating fearful faces, the present study extends these findings, which is particularly noteworthy given that fear has been found to capture attention (e.g. Vuilleumier et al., 2001).

Thus, although emotional faces are highly relevant cues for social interaction (e.g. Blair, 2003; Fridlund & Russel, 2006) and tend to be processed even more automatically than emotional words (Beall & Herbert, 2008), their impact on motor output could disappear when the emotional content of the face is incidental. One possible interpretation of these results is that the processing of emotional information could be non-automatic as suggested in previous studies (Mirabella & Montalti, 2025; Phaf et al., 2014). However, it is important to note that the two experimental conditions differed along several aspects beyond the task-relevance of emotional expressions. In the neutral-versus-emotional condition, participants discriminated between emotional and neutral faces, whereas in the face-gender condition they discriminated between male and female faces. These cognitively different decisions may have contributed to the observed condition effects. In addition, the two conditions differed in stimulus repetition and in the presence of neutral faces, which further limits the extent to which the condition effect can be attributed solely to task-relevance or incidental processing of emotional information. Therefore, the lack of an emotional effect in the face-gender condition cannot be interpreted as arising only from the incidental processing of emotional stimuli, but may also reflect other factors, such as perceptual categorization and response preparation demands.

It is possible that facial expressions were nevertheless processed automatically, but not to an extent strong enough to produce a measurable impact on subsequent behaviour. This interpretation is consistent with the fact that participants were engaged in real approach movements (e.g., single-step initiation) while responding only to symbolic faces (i.e., pictures of human emotional expressions).

### Limitations and future research

Although our findings are consistent with the involvement of approach- and avoidance-related motivational states, these were not directly measured in the present study. Future research should address this issue more explicitly, for instance by incorporating subjective indices of approach–avoidance motivation. Similarly, the perception of threat was not directly assessed. Future studies could include subjective ratings or stimuli that vary in threat intensity to better capture this aspect.

The potentially greater diversity of motor responses observed for happiness, and more broadly for positive emotions, could be further explored by examining inter-individual variability. Specifically, including measures of individual differences in motivational dispositions (e.g., approach-avoidance related traits) may help account for the variability in motor responses across participants.

It is also important to note that backward stepping does not constitute a valid avoidance response. Previous studies using forward and backward stepping paradigms have consistently shown that emotional stimuli modulate forward stepping, but not backward stepping (Coudrat et al., 2017; Stins et al., 2014; Yiou et al., 2011). This asymmetry may reflect the fact that backward stepping is not a true motor counterpart to forward stepping, as it involves greater postural constraints, lacks visual guidance, and is less practiced and functional in everyday behaviour.

Finally, although several statistically significant effects were observed, effect sizes were small (e.g., R² values around 0.001–0.003). These effects indicate that while emotional influences on motor output are real, they are fine and subtle. In addition, the power analysis was conducted for the main hypothesis, focusing on a specific contrast rather than the overall interaction. This analysis relied on a simplified and optimistic scenario, assuming comparable effects across emotional expressions. However, the literature does not provide a clear consensus regarding the effects of fear, which may be weaker from those of other emotions, such as happiness. Consequently, the statistical power may have been overestimated. Together with the relatively small sample size (*n* = 24), this may have limited the ability to detect weaker effects beyond those considered in the a priori power analysis. Therefore, theoretical interpretations should be made cautiously, and future studies with larger samples may help clarify the robustness of these effects.

## Conclusion

The current study provides a better understanding of the links between emotion and action. In particular, the results indicated that approach-related motor responses, assessed through an unambiguous forward-stepping movement, were modulated by emotional faces in the neutral-versus-emotional condition. More precisely, the motor response was stronger (higher anticipatory postural adjustments amplitude) in front of fearful and happy faces than angry faces, and faster (shorter time to reach the forward velocity peak of the centre of mass) to fearful than to angry faces. The way in which the approach-related motor response differ between fearful and angry expressions may be interpreted as reflecting opposing action tendencies elicited by theses emotions. Furthermore, the finding that emotional faces modulated approach-related motor responses only when emotional information was task-relevant may suggest that such processing is not fully automatic. However, the absence of an effect when emotional information was incidental may also be explained by other factors, such as differences in perceptual categorization and response preparation demands.

## Data Availability

Individuals can access detailed description of the strategy and main line of code for power analysis, data file and its codebook through the Open Science Framework website (https://osf.io/a5439/overview). We have no conflicts of interest to disclosure.

## Footnotes

1 KDEF faces: AF01, AF14, AF21, AF22, AF29, AM08, AM10, AM11, AM17, AM22.
